# Effectiveness of mRNA COVID-19 vaccine booster doses against Omicron severe outcomes

**DOI:** 10.1038/s41467-023-36566-1

**Published:** 2023-03-07

**Authors:** Ramandip Grewal, Lena Nguyen, Sarah A. Buchan, Sarah E. Wilson, Sharifa Nasreen, Peter C. Austin, Kevin A. Brown, Deshayne B. Fell, Jonathan B. Gubbay, Kevin L. Schwartz, Mina Tadrous, Kumanan Wilson, Jeffrey C. Kwong

**Affiliations:** 1 Public Health Ontario, Toronto, ON Canada; 2 ICES, Toronto, ON Canada; 3 Dalla Lana School of Public Health, University of Toronto, Toronto, ON Canada; 4 Centre for Vaccine Preventable Diseases, University of Toronto, Toronto, ON Canada; 5 Institute of Health Policy, Management and Evaluation, University of Toronto, Toronto, ON Canada; 6 School of Epidemiology and Public Health, University of Ottawa, Ottawa, ON Canada; 7 Children’s Hospital of Eastern Ontario Research Institute, Ottawa, ON Canada; 8 Department of Laboratory Medicine and Pathobiology, University of Toronto, Toronto, ON Canada; 9 Women’s College Hospital, Toronto, ON Canada; 10 Leslie Dan Faculty of Pharmacy, University of Toronto, Toronto, ON Canada; 11 Department of Medicine, University of Ottawa, Ottawa, ON Canada; 12 Ottawa Hospital Research Institute, Ottawa, ON Canada; 13 Bruyere Research Institute, Ottawa, ON Canada; 14 Department of Family and Community Medicine, University of Toronto, Toronto, ON Canada; 15 University Health Network, Toronto, ON Canada

**Keywords:** Preventive medicine, Epidemiology, Vaccines, SARS-CoV-2

## Abstract

We estimated the effectiveness of booster doses of monovalent mRNA COVID-19 vaccines against Omicron-associated severe outcomes among adults in Ontario, Canada. We used a test-negative design to estimate vaccine effectiveness (VE) against hospitalization or death among SARS-CoV-2-tested adults aged ≥50 years from January 2 to October 1, 2022, stratified by age and time since vaccination. We also compared VE during BA.1/BA.2 and BA.4/BA.5 sublineage predominance. We included 11,160 cases and 62,880 tests for test-negative controls. Depending on the age group, compared to unvaccinated adults, VE was 91–98% 7–59 days after a third dose, waned to 76–87% after ≥240 days, was restored to 92–97% 7–59 days after a fourth dose, and waned to 86–89% after ≥120 days. VE was lower and declined faster during BA.4/BA.5 versus BA.1/BA.2 predominance, particularly after ≥120 days. Here we show that booster doses of monovalent mRNA COVID-19 vaccines restored strong protection against severe outcomes for at least 3 months after vaccination. Across the entire study period, protection declined slightly over time, but waned more during BA.4/BA.5 predominance.

## Abstract (web summary)

This study investigates the protection provided by mRNA COVID-19 vaccine booster doses against Omicron-associated severe disease in adults aged 50 and older. The authors use data from Ontario, Canada, and find that booster doses provide strong protection but that it declined during the period of BA.4/BA.5 predominance.

## Introduction

COVID-19 vaccines first became available in Ontario, Canada in December 2020. Due to concerns about waning protection from the primary series and the emergence of more transmissible SARS-CoV-2 variants, third doses (first boosters) were offered to high-risk groups, including community-dwelling adults aged ≥70 years in November 2021^1^. With the emergence of Omicron, the most transmissible and immune-evasive variant to date^2^, third dose eligibility was expanded to all adults in December 2021^3^. Ontario began offering fourth doses (second boosters) to adults aged ≥60 years in April 2022^4^, and to all adults in July 2022^5^. Booster dose policies differed for residents of long-term care facilities^6^. Bivalent COVID-19 vaccines were introduced to Canadian vaccination programs starting in September 2022 and are now preferred^7,8^, but monovalent vaccines are still authorized for use as boosters and are the products that have been most commonly received to date.

Various Omicron sublineages have circulated during 2022, with BA.1 and BA.2 predominating until June, and BA.4 and BA.5 predominating subsequently. The seroprevalence of prior SARS-CoV-2 infection for the overall population increased substantially in Ontario during the Omicron period, from 6% in early January 2022 to approximately 50% by early July 2022 and 63% by early October 2022^9^. Across Canada, seroprevalence is lower when restricted to adults aged ≥60 years (50% by early October 2022)^9^.

Due to increased transmissibility and immune evasion of emerging Omicron sublineages, more evidence is needed on the long-term effectiveness of booster doses of monovalent mRNA vaccines among older adults to inform planning for subsequent boosters and future shifts in vaccine development. Thus, we sought to estimate vaccine effectiveness (VE) of 2, 3, and 4 doses compared to unvaccinated subjects, and marginal effectiveness of 3 or 4 doses compared to 2 doses, in preventing severe outcomes (hospitalization or death) among community-dwelling adults aged ≥50 years during an Omicron-dominant period. We estimated marginal effectiveness due to concerns about differences between unvaccinated and vaccinated populations. We also sought to determine how VE varied during periods of BA.1/BA.2 versus BA.4/BA.5 predominance.

## Results

We included 11,160 Omicron-associated severe outcomes and 62,880 symptomatic test-negative controls (among 53,369 individuals). Across all study age groups, more cases than controls were male and fewer were unvaccinated (Table 1). Among the 50–59 and 60–69 years age groups, more cases had at least one comorbid condition and were from areas with the lowest incomes. Across all age groups, compared to unvaccinated subjects, more vaccinated subjects had previously received influenza vaccines (Supplementary Tables 2–5). This difference was also seen when comparing subjects with 2 versus 3 or 4 doses (Supplementary Tables 6–9). Among those aged 70–79 years, 36% and 31% of subjects who received a third and fourth dose, respectively, received the mRNA-1273 vaccine. Among subjects aged ≥80 years, 34% and 37% of third and fourth dose recipients received mRNA-1273, respectively.

**Table 1:** Descriptive characteristics of community-dwelling adults aged ≥50 years tested for SARS-CoV-2 and with severe outcomes between January 2, 2022 and October 1, 2022 in Ontario, Canada, comparing Omicron-associated severe outcome cases to SARS-CoV-2 negative controls, stratified by age group

	Age 50–59 years			Age 60–69 years			Age 70–79 years			Age 80+ years		
	SARS-CoV-2 negative controls, *n* (%)^a^	Omicron cases, *n* (%)	SD^b^	SARS-CoV-2 negative controls, *n* (%)^a^	Omicron cases, *n* (%)	SD^b^	SARS-CoV-2 negative controls, *n* (%)^a^	Omicron cases, *n* (%)	SD^b^	SARS-CoV-2 negative controls, *n* (%)^a^	Omicron cases, *n* (%)	SD^b^
Total	25,002	1136		16,139	1926		11,585	2975		10,154	5.123	
Characteristics												
Age (years), mean (standard deviation)	54.45 ± 2.91	55.15 ± 2.86	0.24	63.95 ± 2.85	64.78 ± 2.90	0.29	74.20 ± 2.81	74.79 ± 2.82	0.21	86.27 ± 4.79	86.96 ± 4.93	0.14
Male sex	7370 (29.5%)	659 (58.0%)	0.60	6,396 (39.6%)	1,099 (57.1%)	0.35	5,387 (46.5%)	1,715 (57.6%)	0.22	4,341 (42.8%)	2,858 (55.8%)	0.26
Public health unit region												
Central East	2232 (8.9%)	62 (5.5%)	0.13	1227 (7.6%)	118 (6.1%)	0.06	648 (5.6%)	199 (6.7%)	0.05	387 (3.8%)	312 (6.1%)	0.11
Central West	3794 (15.2%)	224 (19.7%)	0.12	2408 (14.9%)	417 (21.7%)	0.17	1815 (15.7%)	593 (19.9%)	0.11	1609 (15.8%)	950 (18.5%)	0.07
Durham	1552 (6.2%)	48 (4.2%)	0.09	829 (5.1%)	52 (2.7%)	0.13	375 (3.2%)	88 (3.0%)	0.02	133 (1.3%)	121 (2.4%)	0.08
Eastern	1356 (5.4%)	105 (9.2%)	0.15	621 (3.8%)	168 (8.7%)	0.20	347 (3.0%)	265 (8.9%)	0.25	310 (3.1%)	394 (7.7%)	0.21
North	4696 (18.8%)	126 (11.1%)	0.22	3428 (21.2%)	183 (9.5%)	0.33	2533 (21.9%)	249 (8.4%)	0.38	2180 (21.5%)	378 (7.4%)	0.41
Ottawa	599 (2.4%)	57 (5.0%)	0.14	274 (1.7%)	83 (4.3%)	0.15	166 (1.4%)	147 (4.9%)	0.20	187 (1.8%)	240 (4.7%)	0.16
Peel	2266 (9.1%)	125 (11.0%)	0.06	1670 (10.3%)	212 (11.0%)	0.02	1486 (12.8%)	359 (12.1%)	0.02	1613 (15.9%)	579 (11.3%)	0.13
South West	3961 (15.8%)	165 (14.5%)	0.04	2980 (18.5%)	271 (14.1%)	0.12	2425 (20.9%)	409 (13.7%)	0.19	2288 (22.5%)	604 (11.8%)	0.29
Toronto	3122 (12.5%)	143 (12.6%)	0.00	1888 (11.7%)	286 (14.8%)	0.09	1384 (11.9%)	442 (14.9%)	0.09	1234 (12.2%)	1098 (21.4%)	0.25
York	1329 (5.3%)	74 (6.5%)	0.05	760 (4.7%)	129 (6.7%)	0.09	382 (3.3%)	210 (7.1%)	0.17	169 (1.7%)	432 (8.4%)	0.31
Missing	95 (0.4%)	7 (0.6%)	0.03	54 (0.3%)	7 (0.4%)	0.00	24 (0.2%)	14 (0.5%)	0.05	44 (0.4%)	15 (0.3%)	0.02
Household income quintile												
1 (lowest)	4645 (18.6%)	337 (29.7%)	0.26	3552 (22.0%)	577 (30.0%)	0.18	2574 (22.2%)	824 (27.7%)	0.13	2368 (23.3%)	1248 (24.4%)	0.02
2	4841 (19.4%)	221 (19.5%)	0.00	3331 (20.6%)	426 (22.1%)	0.04	2415 (20.8%)	663 (22.3%)	0.04	2291 (22.6%)	1170 (22.8%)	0.01
3	4955 (19.8%)	216 (19.0%)	0.02	3001 (18.6%)	336 (17.4%)	0.03	2215 (19.1%)	552 (18.6%)	0.01	1963 (19.3%)	1024 (20.0%)	0.02
4	5091 (20.4%)	215 (18.9%)	0.04	3053 (18.9%)	332 (17.2%)	0.04	2174 (18.8%)	498 (16.7%)	0.05	1797 (17.7%)	885 (17.3%)	0.01
5 (highest)	5407 (21.6%)	144 (12.7%)	0.24	3152 (19.5%)	250 (13.0%)	0.18	2168 (18.7%)	419 (14.1%)	0.13	1687 (16.6%)	785 (15.3%)	0.04
Missing	63 (0.3%)	3 (0.3%)	0.00	50 (0.3%)	5 (0.3%)	0.01	39 (0.3%)	19 (0.6%)	0.04	48 (0.5%)	11 (0.2%)	0.04
Essential workers quintile												
1 (0%–32.5%)	3941 (15.8%)	110 (9.7%)	0.18	2266 (14.0%)	209 (10.9%)	0.10	1782 (15.4%)	382 (12.8%)	0.07	1699 (16.7%)	865 (16.9%)	0.00
2 (32.5%–42.3%)	5669 (22.7%)	236 (20.8%)	0.05	3354 (20.8%)	361 (18.7%)	0.05	2368 (20.4%)	581 (19.5%)	0.02	2230 (22.0%)	1085 (21.2%)	0.02
3 (42.3%–49.8%)	5429 (21.7%)	236 (20.8%)	0.02	3535 (21.9%)	420 (21.8%)	0.00	2503 (21.6%)	621 (20.9%)	0.02	2264 (22.3%)	1087 (21.2%)	0.03
4 (50.0%–57.5%)	5157 (20.6%)	235 (20.7%)	0.00	3440 (21.3%)	437 (22.7%)	0.03	2429 (21.0%)	654 (22.0%)	0.02	1938 (19.1%)	1112 (21.7%)	0.07
5 (57.5%–100%)	4595 (18.4%)	307 (27.0%)	0.21	3403 (21.1%)	483 (25.1%)	0.09	2437 (21.0%)	714 (24.0%)	0.07	1962 (19.3%)	953 (18.6%)	0.02
Missing	211 (0.8%)	12 (1.1%)	0.02	141 (0.9%)	16 (0.8%)	0.00	66 (0.6%)	23 (0.8%)	0.02	61 (0.6%)	21 (0.4%)	0.03
Persons per dwelling quintile												
1 (0–2.1)	4518 (18.1%)	263 (23.2%)	0.13	3647 (22.6%)	489 (25.4%)	0.07	3131 (27.0%)	805 (27.1%)	0.00	3268 (32.2%)	1362 (26.6%)	0.12
2 (2.2–2.4)	5220 (20.9%)	235 (20.7%)	0.00	3682 (22.8%)	396 (20.6%)	0.05	2733 (23.6%)	577 (19.4%)	0.10	2344 (23.1%)	958 (18.7%)	0.11
3 (2.5–2.6)	3480 (13.9%)	168 (14.8%)	0.02	2182 (13.5%)	281 (14.6%)	0.03	1505 (13.0%)	411 (13.8%)	0.02	1345 (13.2%)	732 (14.3%)	0.03
4 (2.7–3.0)	5747 (23.0%)	225 (19.8%)	0.08	3269 (20.3%)	359 (18.6%)	0.04	2308 (19.9%)	561 (18.9%)	0.03	1822 (17.9%)	1084 (21.2%)	0.08
5 (3.1–5.7)	5824 (23.3%)	233 (20.5%)	0.07	3203 (19.8%)	382 (19.8%)	0.00	1844 (15.9%)	595 (20.0%)	0.11	1303 (12.8%)	962 (18.8%)	0.16
Missing	213 (0.9%)	12 (1.1%)	0.02	156 (1.0%)	19 (1.0%)	0.00	64 (0.6%)	26 (0.9%)	0.04	72 (0.7%)	25 (0.5%)	0.03
Self-identified visible minority quintile												
1 (0.0%–2.2%)	5864 (23.5%)	247 (21.7%)	0.04	4054 (25.1%)	392 (20.4%)	0.11	2811 (24.3%)	605 (20.3%)	0.09	2126 (20.9%)	865 (16.9%)	0.10
2 (2.2%–7.5%)	5445 (21.8%)	181 (15.9%)	0.15	3624 (22.5%)	345 (17.9%)	0.11	2675 (23.1%)	548 (18.4%)	0.12	2305 (22.7%)	902 (17.6%)	0.13
3 (7.5%–18.7%)	4387 (17.5%)	192 (16.9%)	0.02	2771 (17.2%)	356 (18.5%)	0.03	2039 (17.6%)	514 (17.3%)	0.01	2150 (21.2%)	955 (18.6%)	0.06
4 (18.7%–43.5%)	4174 (16.7%)	231 (20.3%)	0.09	2488 (15.4%)	347 (18.0%)	0.07	1963 (16.9%)	555 (18.7%)	0.04	1894 (18.7%)	1069 (20.9%)	0.06
5 (43.5%–100%)	4921 (19.7%)	273 (24.0%)	0.11	3061 (19.0%)	470 (24.4%)	0.13	2031 (17.5%)	731 (24.6%)	0.17	1618 (15.9%)	1311 (25.6%)	0.24
Missing	211 (0.8%)	12 (1.1%)	0.02	141 (0.9%)	16 (0.8%)	0.00	66 (0.6%)	22 (0.7%)	0.02	61 (0.6%)	21 (0.4%)	0.03
Receipt of 2019–2020 and/or 2020–2021 influenza vaccination	8842 (35.4%)	311 (27.4%)	0.17	8064 (50.0%)	777 (40.3%)	0.19	8094 (69.9%)	1594 (53.6%)	0.34	7573 (74.6%)	3249 (63.4%)	0.24
Prior positive SARS-CoV-2 test	1674 (6.7%)	29 (2.6%)	0.20	789 (4.9%)	21 (1.1%)	0.22	345 (3.0%)	35 (1.2%)	0.13	248 (2.4%)	43 (0.8%)	0.13
Number of SARS-CoV-2 tests within 3 months prior to December 14, 2020												
0	15,958 (63.8%)	957 (84.2%)	0.48	11,629 (72.1%)	1660 (86.2%)	0.35	9622 (83.1%)	2662 (89.5%)	0.19	8676 (85.4%)	4596 (89.7%)	0.13
1	4364 (17.5%)	117 (10.3%)	0.21	2337 (14.5%)	187 (9.7%)	0.15	1341 (11.6%)	225 (7.6%)	0.14	953 (9.4%)	361 (7.0%)	0.09
≥2	4680 (18.7%)	62 (5.5%)	0.42	2173 (13.5%)	79 (4.1%)	0.34	622 (5.4%)	88 (3.0%)	0.12	525 (5.2%)	166 (3.2%)	0.10
Any comorbidity	14,709 (58.8%)	893 (78.6%)	0.44	12,236 (75.8%)	1665 (86.4%)	0.27	10,362 (89.4%)	2791 (93.8%)	0.16	9826 (96.8%)	4995 (97.5%)	0.04
Receipt of home care services												
None	24,611 (98.4%)	1058 (93.1%)	0.27	15,486 (96.0%)	1791 (93.0%)	0.13	10,683 (92.2%)	2726 (91.6%)	0.02	8861 (87.3%)	4537 (88.6%)	0.04
Short stay	279 (1.1%)	41 (3.6%)	0.16	402 (2.5%)	64 (3.3%)	0.05	459 (4.0%)	104 (3.5%)	0.02	512 (5.0%)	162 (3.2%)	0.09
Long stay	96 (0.4%)	32–36 (2.8–3.2%)	0.20	217 (1.3%)	60 (3.1%)	0.12	394 (3.4%)	135 (4.5%)	0.06	734 (7.2%)	401 (7.8%)	0.02
Palliative	16 (0.1%)	≤5 (≤0.4%)	0.05	34 (0.2%)	11 (0.6%)	0.06	49 (0.4%)	10 (0.3%)	0.01	47 (0.5%)	23 (0.4%)	0.00
Unvaccinated^c^	944 (3.8%)	416 (36.6%)	0.90	840 (5.2%)	695 (36.1%)	0.83	572 (4.9%)	880 (29.6%)	0.69	538 (5.3%)	1136 (22.2%)	0.51
Time since second dose^c^												
7–59 days	119 (0.5%)	10 (0.9%)	0.05	60 (0.4%)	10 (0.5%)	0.02	25 (0.2%)	6 (0.2%)	0.00	27 (0.3%)	10 (0.2%)	0.01
60–119 days	396 (1.6%)	37 (3.3%)	0.11	149 (0.9%)	27 (1.4%)	0.04	82 (0.7%)	27 (0.9%)	0.02	32 (0.3%)	34 (0.7%)	0.05
120–179 days	1066 (4.3%)	65 (5.7%)	0.07	544 (3.4%)	107 (5.6%)	0.11	205 (1.8%)	112 (3.8%)	0.12	135 (1.3%)	125 (2.4%)	0.08
180–239 days	2246 (9.0%)	136 (12.0%)	0.10	1,230 (7.6%)	206 (10.7%)	0.11	538 (4.6%)	332 (11.2%)	0.24	423 (4.2%)	542 (10.6%)	0.25
240–299 days	848 (3.4%)	55 (4.8%)	0.07	524 (3.2%)	108 (5.6%)	0.11	357 (3.1%)	133 (4.5%)	0.07	290 (2.9%)	180 (3.5%)	0.04
≥300 days	1,215 (4.9%)	86 (7.6%)	0.11	778 (4.8%)	139 (7.2%)	0.10	447 (3.9%)	163 (5.5%)	0.08	471 (4.6%)	363 (7.1%)	0.10
Time since third dose^c^												
0–6 days	402 (1.6%)	8 (0.7%)	0.08	233 (1.4%)	14 (0.7%)	0.07	109 (0.9%)	30 (1.0%)	0.01	65 (0.6%)	49 (1.0%)	0.04
7–59 days	5681 (22.7%)	68 (6.0%)	0.49	3650 (22.6%)	94 (4.9%)	0.53	2101 (18.1%)	182 (6.1%)	0.37	1496 (14.7%)	299 (5.8%)	0.30
60–119 days	4830 (19.3%)	80 (7.0%)	0.37	2892 (17.9%)	145 (7.5%)	0.32	1994 (17.2%)	243 (8.2%)	0.27	1902 (18.7%)	503 (9.8%)	0.26
120–179 days	3583 (14.3%)	50 (4.4%)	0.35	2104 (13.0%)	117 (6.1%)	0.24	1699 (14.7%)	246 (8.3%)	0.20	1400 (13.8%)	509 (9.9%)	0.12
180–239 days	2086 (8.3%)	73 (6.4%)	0.07	1116 (6.9%)	128 (6.6%)	0.01	816 (7.0%)	226 (7.6%)	0.02	685 (6.7%)	448 (8.7%)	0.07
≥240 days	1040 (4.2%)	40 (3.5%)	0.03	479 (3.0%)	46 (2.4%)	0.04	343 (3.0%)	109 (3.7%)	0.04	324 (3.2%)	252 (4.9%)	0.09
Time since fourth dose^c^												
0–6 days	32 (0.1%)	≤5 (≤0.4%)^d^	0.01	95 (0.6%)	≤5 (≤0.3%)^d^	0.08	103 (0.9%)	10 (0.3%)	0.07	83 (0.8%)	29 (0.6%)	0.03
7–59 days	357 (1.4%)	≤5 (≤0.4%)^d^	0.11	730 (4.5%)	28 (1.5%)	0.18	979 (8.5%)	85 (2.9%)	0.24	905 (8.9%)	152 (3.0%)	0.25
60–119 days	119 (0.5%)	≤5 (≤0.4%)^d^	0.03	511 (3.2%)	40 (2.1%)	0.07	837 (7.2%)	106 (3.6%)	0.16	835 (8.2%)	255 (5.0%)	0.13
≥120 days	38 (0.2%)	≤5 (≤0.4%)^d^	0.02	204 (1.3%)	17-21 (0.9–1.1%)^d^	0.02	378 (3.3%)	85 (2.9%)	0.02	543 (5.3%)	237 (4.6%)	0.03

^*^Note, not unique by person; individuals may be included more than once.

^a^Proportion reported, unless stated otherwise.

^b^SD = standardized difference. Standardized differences of >0.10 are considered clinically relevant. Comparing Omicron cases to test-negative controls.

^c^Sum of all rows (unvaccinated and vaccinated) equals 100%.

^d^Due to institutional privacy policies, any cells ≤5 (except for missing values) must be suppressed and ranges must be provided for complementary cells to prevent back calculation.

### Vaccine effectiveness and marginal effectiveness

**Fig. 1:**
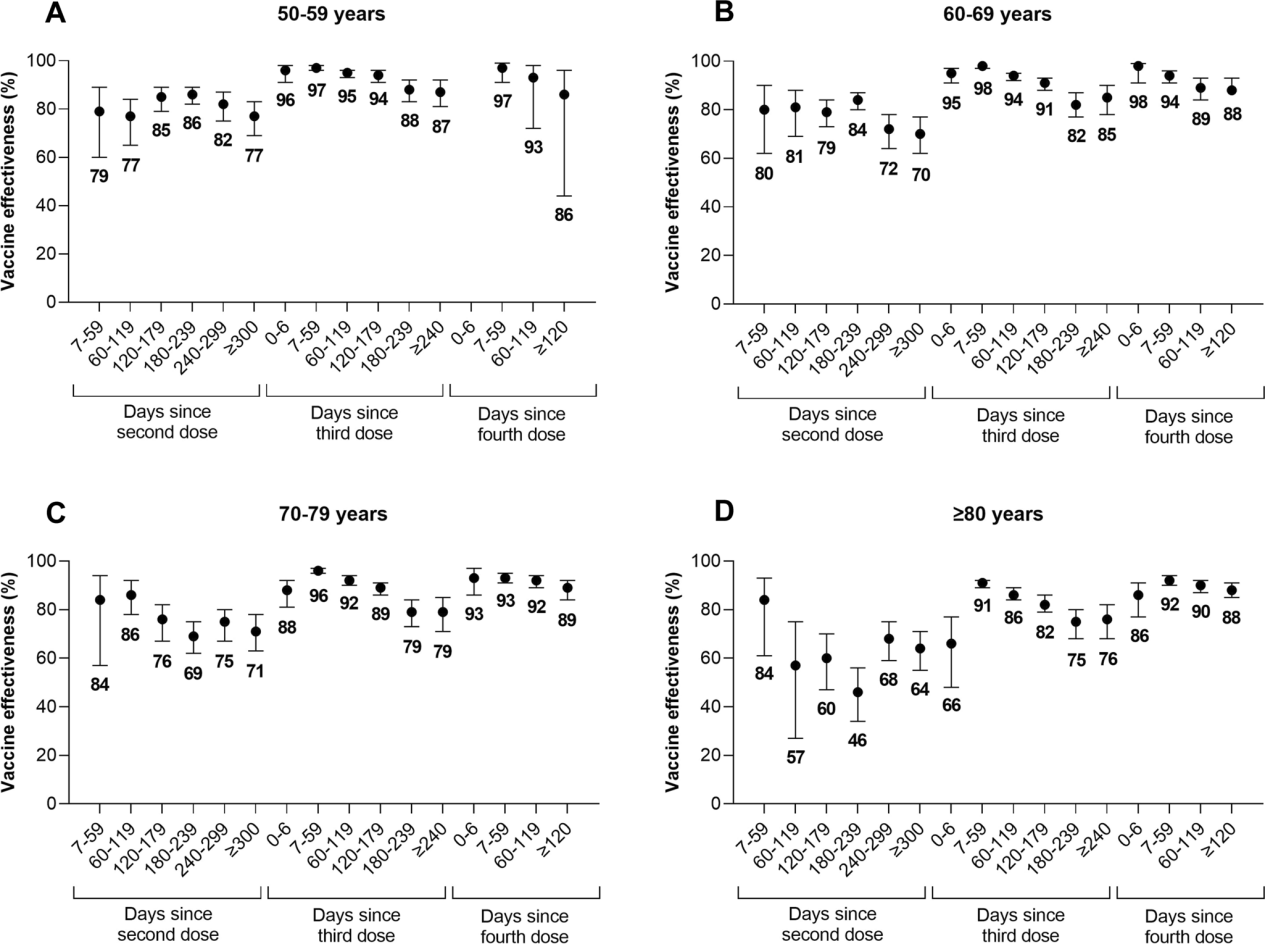
Vaccine effectiveness and 95% confidence intervals by time since vaccination. Vaccine effectiveness (presented as proportions out of 100 percentage points) and 95% confidence intervals of 2, 3, and 4 doses of monovalent mRNA COVID-19 vaccines against Omicron-associated severe outcomes by time since vaccination among community-dwelling adults aged (**A**) 50–59 years, (**B**) 60–69 years, (**C**) 70–79 years, and (**D**) ≥ 80 years in Ontario, Canada, compared to unvaccinated adults (Note: Estimates were not reported if they were unstable [i.e., 95% confidence interval width exceeded 100 percentage points]). Please see Supplementary Table 10 for all estimates.

Compared to unvaccinated subjects, VE against severe disease increased shortly after receipt of booster doses but subsequently declined over time (Fig. 1, Supplementary Tables 10–11). For example, among subjects aged 70–79 years, VE decreased from: 84% (95% CI, 57–94%) 7–59 days after a second dose to 71% (95% CI, 63–78%) after ≥300 days; 96% (95% CI, 95–97%) 7–59 days after a third dose to 79% (95% CI, 71–85%) after ≥240 days; and 93% (95% CI, 91–95%) 7–59 days after a fourth dose to 89% (95% CI, 84–92%) after ≥120 days. The decline in VE after a third dose appeared to plateau after 180 days. VE was generally lower with increasing age.

**Fig. 2:**
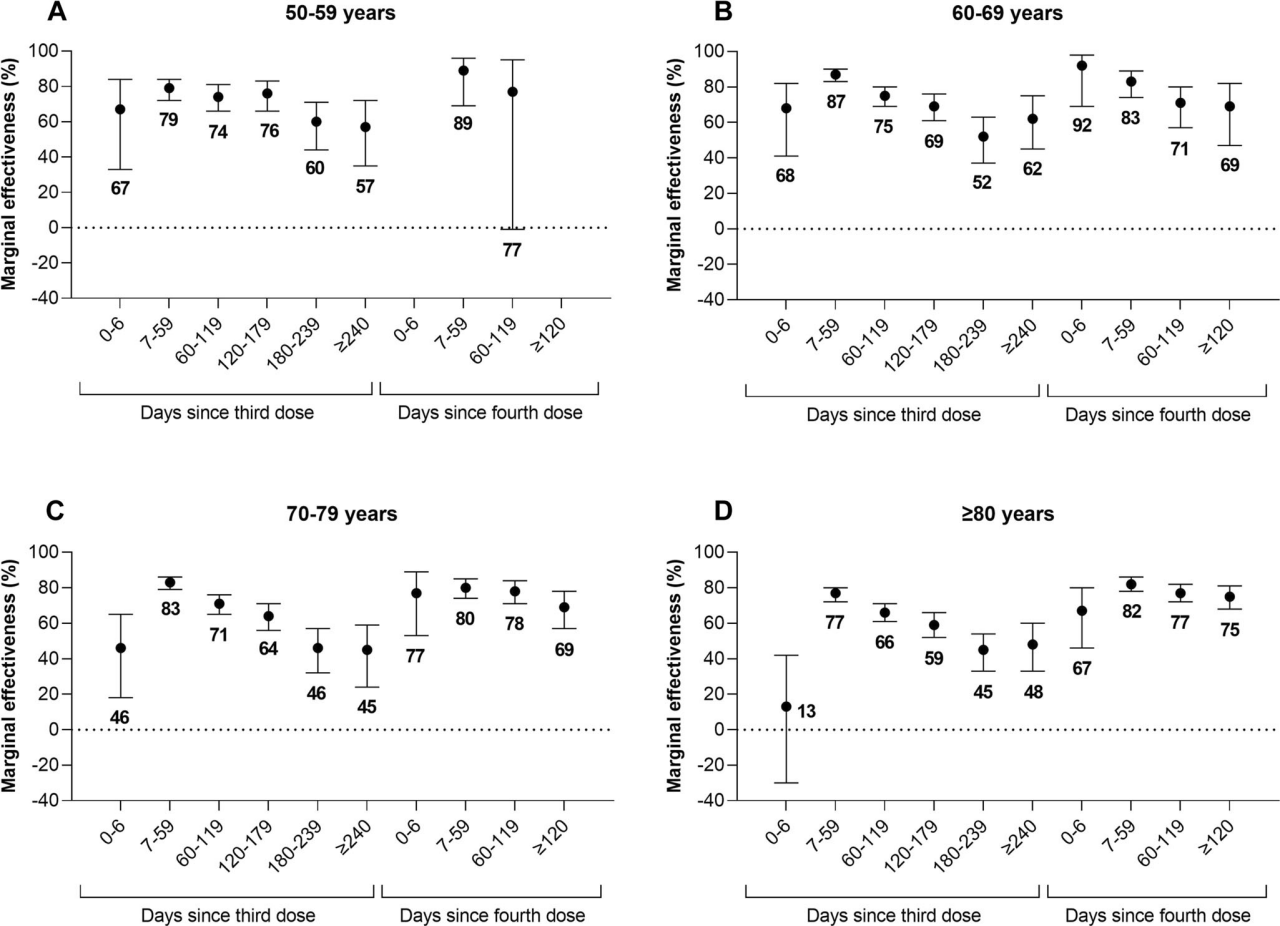
Marginal effectiveness and 95% confidence intervals by time since vaccination. Marginal effectiveness (presented as proportions out of 100 percentage points) and 95% confidence intervals of a third and fourth dose of monovalent mRNA COVID-19 vaccines against Omicron-associated severe outcomes by time since vaccination among community-dwelling adults aged (**A**) 50–59 years, (**B**) 60–69 years, (**C**) 70–79 years, and (**D**) ≥80 years in Ontario, Canada, compared to adults who received 2 doses. (Note: Estimates were not reported if they were unstable [i.e., 95% confidence interval width exceeded 100 percentage points]). Please see Supplementary Table 12 for all estimates.

Marginal effectiveness peaked 7–59 days after third and fourth doses and declined over time. Once again, among subjects aged 70–79 years, compared to 2 doses (median 218 days since a second dose), the marginal effectiveness of a third dose decreased from 83% (95% CI, 79–86%) 7–59 days after a third dose to 45% (95% CI, 24–59%) after ≥240 days and the marginal effectiveness of a fourth dose decreased from 80% (95% CI, 74–85%) 7–59 days after a fourth dose to 69% (95% CI, 57–78%) after ≥120 days (Fig. 2, Supplementary Table 12).

### Additional analyses

**Fig. 3:**
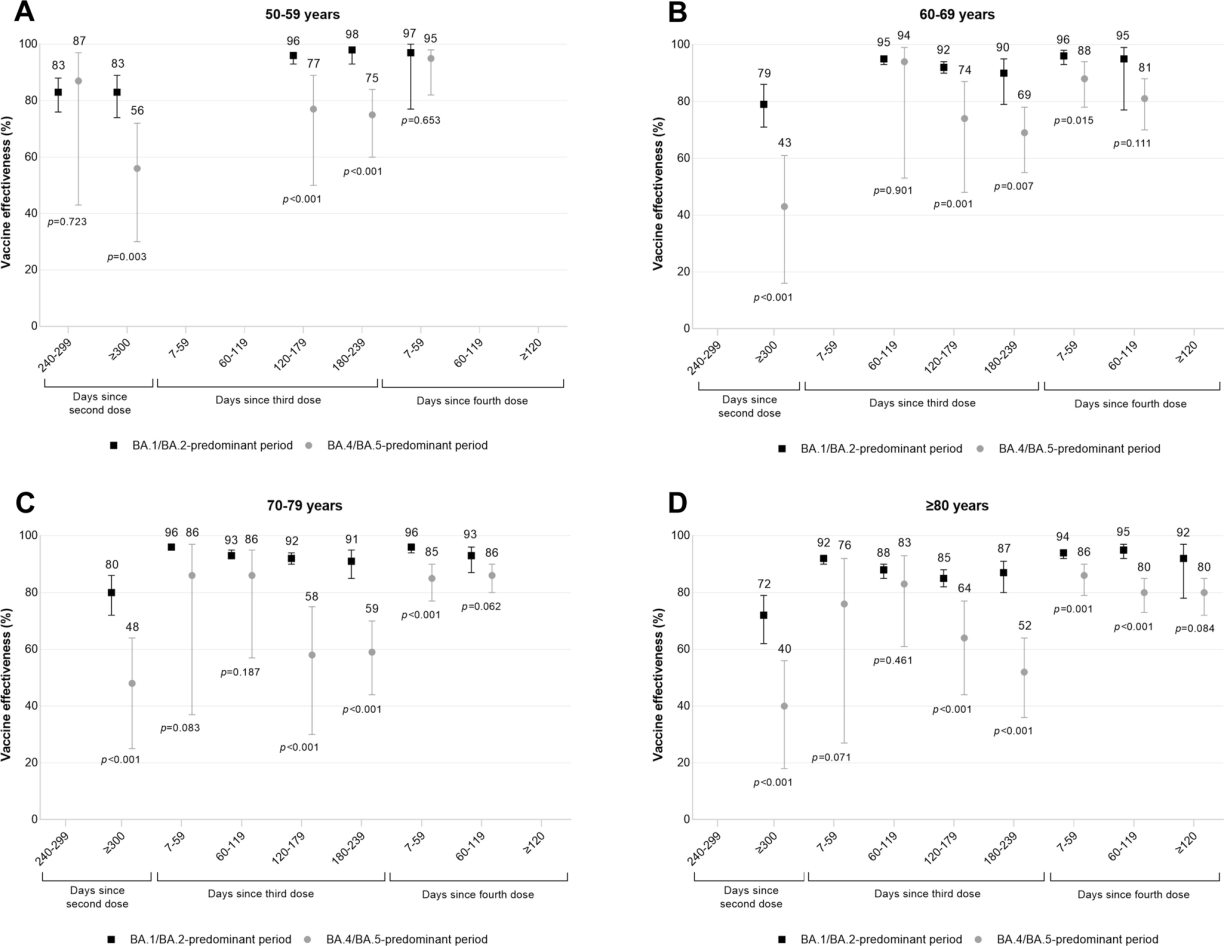
Vaccine effectiveness and 95% confidence intervals, during periods of BA.1/BA.2 (January 2 to July 2, 2022) and BA.4/BA.5 (July 3 to October 1, 2022) predominance, by time since vaccination. Vaccine effectiveness (presented as proportions out of 100 percentage points) and 95% confidence intervals against Omicron-associated severe outcomes among community-dwelling adults aged (**A**) 50–59 years, (**B**) 60–69 years, (**C**) 70–79 years, and (**D**) ≥80 years in Ontario, Canada, comparing those who received ≥2 doses of monovalent mRNA COVID-19 vaccines to those who received none, by age and time since vaccination, during periods of BA.1/BA.2 (January 2 to July 2, 2022) and BA.4/BA.5 (July 3 to October 1, 2022) predominance. (Note: Estimates were not reported if they were unstable [i.e., 95% confidence interval width exceeded 100 percentage points] for either period). Please see Supplementary Table 13 for all estimates.

VE estimates were lower during the BA.4/BA.5-predominant period compared to the BA.1/BA.2-predominant period, with differences widening as time since vaccination increased (Fig. 3, Supplementary Table 13). For example, among subjects aged 70–79 years, VE 7–59 days after a third dose was 96% (95% CI, 96–97%) (median 34 days since a third dose) during the BA.1/BA.2-predominant period compared to 86% (95% CI, 37–97%) (median 41 days since a third dose) during the BA.4/BA.5-predominant period (*p* = 0.08 for the between-period interaction), whereas VE 180–239 days after a third dose was 91% (95% CI, 85–95%) (median 189 days since a third dose) during the BA.1/BA.2-predominant period compared to 59% (95% CI, 44–70%) (median 214 days since a third dose) during the BA.4/BA.5-predominant period (*p* < 0.001 for the between-period interaction).

When Paxlovid recipients were removed from the analysis, VE estimates were nearly identical to those from the main analysis (Supplementary Table 14).

## Discussion

Among community-dwelling adults aged ≥50 years in Ontario, VE against Omicron-associated severe outcomes increased with booster doses of monovalent mRNA COVID-19 vaccines, but protection waned over time after each dose. Third doses continued to provide strong protection (85–87%) against severe outcomes among subjects aged 50–69 years even 8 months after vaccination, but lower protection (76–79%) among those aged ≥70 years. Fourth doses restored waning of protection from third doses and continued to provide strong protection (86–89%) 4 months after vaccination for all age groups. However, VE in the BA.4/BA.5-predominant period was lower than during the BA.1/BA.2-predominant period across the same time intervals after vaccination, especially with increasing time since vaccination.

Comparisons with other jurisdictions are challenging due to heterogeneity in study designs, population characteristics, outcomes and exposures, vaccines, and observation periods. Our fourth dose VE estimates were slightly higher than those observed in the United States, where fourth dose VE against hospitalizations was 80% (95%CI, 71–85%) after ≥7 days among adults aged ≥50 years^10^. Studies from Israel found that waning of protection against severe outcomes was significantly slower than against infection and that marginal effectiveness of booster doses against infection waned faster after fourth doses compared to third doses^11–13^. They were unable to determine if trends were similar for severe outcomes due to the short follow-up period. In our study, the waning of protection against severe outcomes observed ≥120 days after a fourth dose was comparable to that seen 120–179 days after a third dose. Although differences in timing of vaccination within those time periods may influence VE estimates, the median time since vaccination was 141–145 days (depending on the age group) for the 120–179 days post third dose group and 140–147 days for the ≥120 days post fourth dose group, suggesting that waning of protection after a fourth dose may follow a similar trajectory as after a third dose.

Available evidence on VE of monovalent COVID-19 vaccines against severe outcomes among adults aged ≥18 years caused by the BA.4/BA.5 Omicron sublineages varies. In the UK, compared to a second dose, marginal effectiveness of a third or fourth dose against BA.4/BA.5- versus BA.2-related hospitalizations was similar using the same time intervals since vaccination^14^. Similarly, VE of a third dose against hospitalizations was comparable between BA.1/BA.2-predominant and BA.4/BA.5-predominant periods in South Africa^15^. Conversely, in Portugal, 3-dose protection against severe outcomes was lower among BA.5 versus BA.2 cases^16^. A study among Kaiser Permanente members found that VE of third and fourth doses against BA.4/BA.5-related hospitalizations was lower compared to BA.1/BA.2-related hospitalizations, whereas another study among individuals admitted to IVY Network hospitals saw this difference for a third dose but not for 2 or 4 doses^17,18^. The IVY Network study reported 3-dose VE of 79% (95% CI, 74–84%) and 60% (95% CI, 12–81%), respectively, during the BA.1/BA.2-predominant versus BA.4/BA.5-predominant periods 7–120 days after vaccination^18^.

Potential explanations for lower VE during the period of BA.4/BA.5-predominance compared to BA.1/BA.2-predominance include longer intervals between booster dose receipt and outcomes, increased incidence of undocumented infections, and increased BA.4/BA.5 immune evasion^17^. In our study, differences in the median numbers of days since booster receipt between the BA.1/BA.2-predominant versus BA.4/BA.5-predominant periods were always <30 days, so longer post-booster follow-up during the BA.4/BA.5-predominant period was unlikely to be a major contributor to the large observed differences in VE. VE may be underestimated in the setting of undocumented infections if unvaccinated individuals are more likely to be infected than vaccinated individuals because the former will have infection-induced immunity, and the extent of VE underestimation may increase as prior infections become more prevalent in the population. During the BA.1/BA.2-predominant period, infection-acquired seroprevalence in Ontario in the overall population increased from 6% to 50% and subsequently increased from 50% to 63% during the BA.4/BA.5-predominant period^9^. Seroprevalence was lower among adults aged ≥60 years^9^. However, we noted that VE declined considerably faster as time since vaccination increased during the relatively brief BA.4/BA.5-predominant period (only 3 months) compared to the BA.1/BA.2-predominant period, suggesting that bias from undocumented prior infections is unlikely to account entirely for the differences. Therefore, among these potential explanations, increased immune evasion by BA.4/BA.5 sublineages is likely the largest contributor to these differences in VE.

Based on the evidence to date, the level of protection offered by bivalent vaccines remains unclear. Findings from a phase 2-3 trial suggest that Moderna’s bivalent vaccine elicits higher titres of neutralizing antibodies against Omicron sublineages BA.1 and BA.4/BA.5 compared to Moderna’s ancestral monovalent vaccine^19^. In contrast, two observational immunogenicity studies found the Pfizer and Moderna bivalent vaccines elicited similar levels of neutralizing antibodies against BA.4/BA.5 as the ancestral monovalent vaccines^20,21^.

This study had some limitations. First, data on rapid antigen tests were not available, and this was the main source of testing after December 31, 2021, when eligibility for RT-PCR testing was restricted in Ontario to individuals considered at high risk of acquiring SARS-CoV-2^22^. Thus, while we adjusted for prior SARS-CoV-2 infections documented by RT-PCR, we could not account for prior infections confirmed only by rapid antigen tests. This could bias VE estimates downward or upward depending on whether unvaccinated or vaccinated individuals are more likely to have prior undocumented infections. Second, because whole genome sequencing was not performed on all cases, we were unable to estimate VE against BA.1, BA.2, BA.4, and BA.5 separately but instead combined BA.1 and BA.2 during one period and BA.4 and BA.5 during another period based on when each grouping circulated. Last, there remains the potential for residual confounding since we were limited to the covariates available in the databases used. Unmeasured differences between unvaccinated and vaccinated individuals may introduce bias, but the consistency between the VE and marginal effectiveness estimates is reassuring. A significant strength of our study is the length of the follow-up period, allowing us to estimate VE ≥ 4 months after fourth doses. Also, unlike most other studies, we stratified our analyses by age group, which provides more refined VE estimates for decision-making.

Our findings suggest that while booster doses of monovalent mRNA COVID-19 vaccines initially restore strong protection against Omicron-associated hospitalizations and death among community-dwelling older adults and then subsequently wane over time, much uncertainty remains. Although protection remained strong 4 months after a fourth dose for all age groups, whether waning increases past this period remains unknown, and combined with the evidence of reduced VE against BA.4/BA.5 sublineages and the possibility that vaccines could be even less effective against newly emerging sublineages such as BQ.1.1 and XBB, subsequent boosters and other measures (e.g., face masks, improved ventilation, filtration of indoor air) may be needed to mitigate the impact of Omicron and future SARS-CoV-2 variants. It will be important to continue monitoring VE given the scarcity of VE data against BA.4/BA.5, newly emerging sublineages, and the introduction of bivalent vaccines.

## Methods

### Study design, setting, population, and data sources

Similar to past studies on COVID-19 VE in Ontario^6,23,24^, we applied a test-negative design to provincial SARS-CoV-2 laboratory testing, COVID-19 surveillance, COVID-19 vaccination, and health administrative datasets. These datasets were linked using unique encoded identifiers and analyzed at ICES (formerly the Institute of Clinical Evaluative Sciences). The use of the data in this study is authorized under section 45 of Ontario’s Personal Health Information Protection Act, and does not require review by a research ethics board.

We included community-dwelling adults aged ≥50 years who had ≥1 reverse-transcription polymerase chain reaction (RT-PCR) test for SARS-CoV-2 between January 2, 2022 and October 1, 2022. We excluded immunocompromised individuals (*n* = 11,514) and those who received a bivalent mRNA vaccine (*n* = 2433), Ad26.COV2 (*n* = 83), or >1 dose of ChAdOx1-S (*n* = 1043) by the index date (Supplementary Fig. 1). Approximately 95% of individuals received mRNA vaccines (mRNA-1273 or BNT162b2) for all doses. Omicron represented nearly 100% of all positive samples by late January 2022^25–27^. Delta (B.1.617.2) cases identified using whole genome sequencing or based on an S-gene target positive screening result before January 24, 2022 (*n* = 72) were excluded.

### Outcome and sampling strategy

The case definition was COVID-19-associated hospitalization or death due to, or partially due to, COVID-19. The public health COVID-19 surveillance database data entry guidelines specify that hospitalization data should only be entered for cases who received treatment for COVID-19 while in hospital and/or if their length of stay was extended due to COVID-19^28^. We excluded hospitalizations when specimen collection occurred >3 days after admission and those flagged as being nosocomial. We sampled cases and controls by week of test, thus individuals could enter the study repeatedly, but once an individual became a case, they could not re-enter the study. We employed this sampling strategy to ensure the distribution of time of testing was consistent between cases and controls. Controls had to be symptomatic (Supplementary Text) and test negative for SARS-CoV-2, but may or may not have had a severe outcome. The index date was the earliest of specimen collection, hospitalization, or death.

### COVID-19 vaccination

We classified community-dwelling adults by the number of doses received and time since most recent vaccination relative to the index date. For 2, 3, and 4 doses, we explored up to ≥300 days, ≥240 days, and ≥120 days post-vaccination, respectively. For booster doses of mRNA-1273, a half dose (50 mcg) was recommended for those younger than 70 years and a full dose (100 mcg) for those aged ≥70 years^29^.

### Statistical analysis

We used means and proportions to describe our sample by comparing: 1) test-negative symptomatic controls to test-positive Omicron cases who were hospitalized or died; 2) unvaccinated subjects to those who had received 2, 3, or 4 doses; and 3) subjects who had received 2 doses (≥7 days ago) to those who had received 3 or 4 doses. We used standardized differences (SD) to quantify the differences between groups.

Stratified by age group (50–59, 60–69, 70–79, ≥80 years), we used multivariable logistic regression to compare the odds of vaccination in cases to test-negative controls while adjusting for sex, age (continuous), public health unit region, four area-level variables representing different socio-demographic characteristics (household income quintile, essential worker quintile, persons per dwelling quintile, self-identified visible minority quintile), influenza vaccination during 2019–2020 or 2020–2021 (proxy for health behaviors), SARS-CoV-2 infection >90 days prior, number of SARS-CoV-2 tests within 3 months prior to December 14, 2020 (proxy for healthcare workers), comorbidities, receipt of home care services, and week of test (Supplementary Table 1). We estimated the logistic regression models using generalized estimating equations (GEE) with an exchangeable correlation structure since controls could be in a model more than once (13% of controls) leading to non-independence of observations. We calculated both VE and marginal effectiveness using the formula: (1-adjusted odds ratio)*100%.

**Fig. 4:**
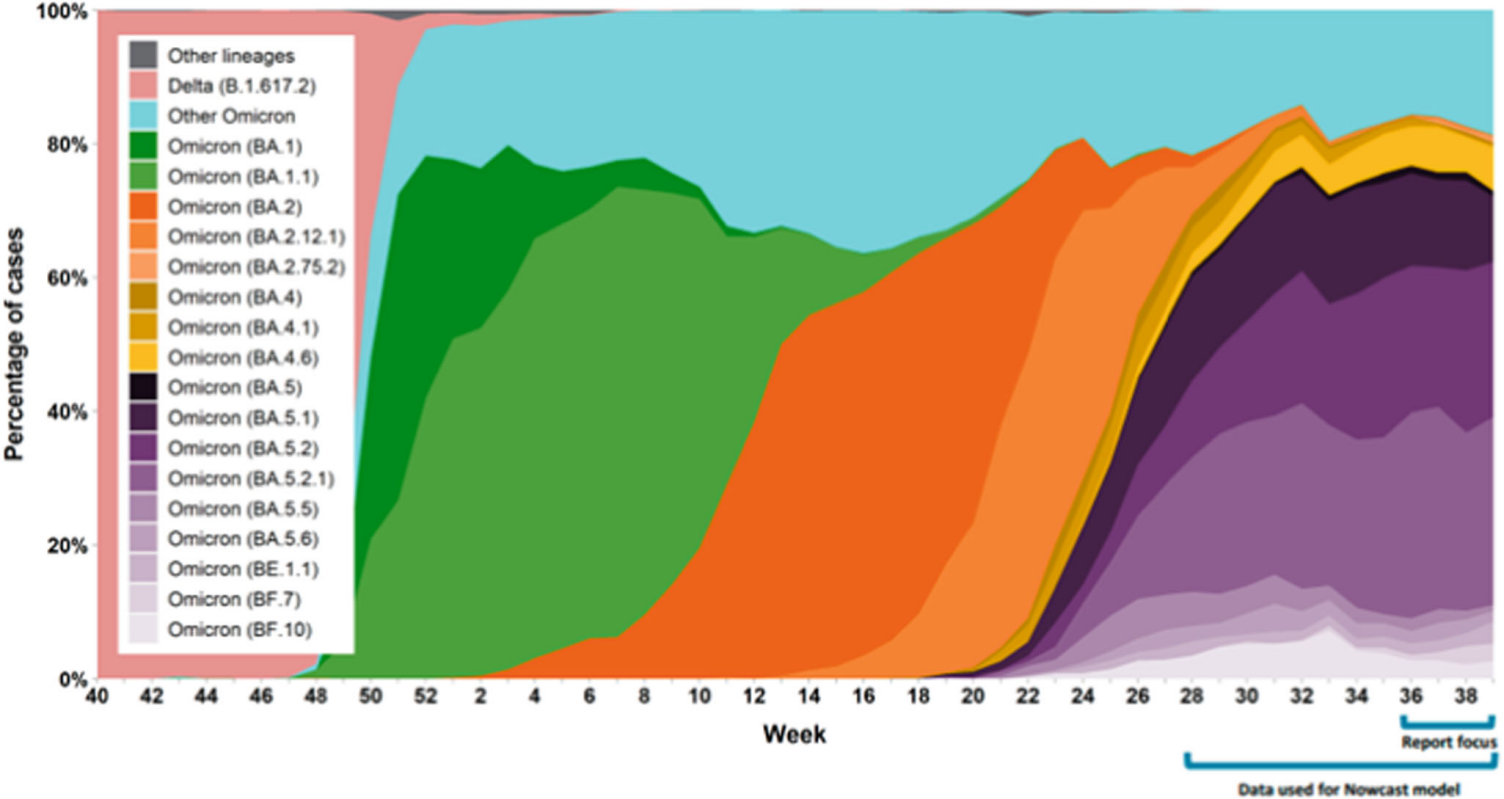
Percentage of COVID-19 cases by the most prevalent lineages and week in Ontario from October 3, 2021 to October 1, 2022. Percentage of COVID-19 cases by the most prevalent lineages and week, representative surveillance, Ontario, October 3, 2021 to October 1, 2022^27^. Each color represents a different lineage or sublineage of Omicron. Note: results may not be representative of Ontario overall, particularly in earlier weeks. Week was assigned based on earliest date available for a sample. If more than one sample was sequenced for a case, the most recent sample was included. Results for recent weeks are incomplete as not all sequencing and bioinformatics analyses were complete at the time of data extraction. Data sources: Public Health Ontario, Hospital for Sick Children, Kingston Health Sciences Centre, Shared Hospital Laboratory, Hamilton Regional Laboratory Medicine Program.

To examine VE against various Omicron sublineages, we included in our multivariable models an interaction term for time period (BA.1/BA.2-predominant period: January 2, 2022 to July 2, 2022; BA.4/BA.5-predominant period: July 3, 2022 to October 1, 2022) (Fig. 4)^27^. Among these sublineages, the distributions were approximately 50% BA.1 and 50% BA.2 during the BA.1/BA.2-predominant period and 10% BA.4 and 90% BA.5 during the BA.4/BA.5-predominant period^27^. GEE methods were not used in this analysis due to issues with convergence. For the estimates that did converge, GEE and non-GEE estimates and 95% confidence intervals were nearly identical. Additionally, as a sensitivity analysis, we excluded all subjects who had been prescribed Paxlovid within 14 days prior to their index date (*n* = 177) to determine whether treatment impacted VE estimates.

We used SAS version 9.1 (SAS Institute Inc., Cary, NC) for all analyses. All tests were 2-sided and we used *p* < 0.05 as the level of significance. A SDs ≥0.1 were considered clinically relevant.

### Reporting summary

Further information on research design is available in the Nature Portfolio Reporting Summary linked to this article.

## Supplementary information

Supplementary Information

Peer Review File

Reporting Summary

## Data Availability

The dataset from this study is held securely in coded form at ICES. While legal data sharing agreements between ICES and data providers (e.g., healthcare organizations and government) prohibit ICES from making the dataset publicly available, access may be granted to those who meet prespecified criteria for confidential access, available at https://www.ices.on.ca/DAS (email: das@ices.on.ca).

## Code availability

The full dataset creation plan and underlying analytic code are available from the authors upon request, understanding that the computer programs may rely upon coding templates or macros that are unique to ICES and are therefore either inaccessible or may require modification.

## References

[1] Government of Ontario. Ontario expanding booster eligibility to more Ontarians. https://news.ontario.ca/en/release/1001100/ontario-expanding-booster-eligibility-to-more-ontarians (2020).

[2] Andrews N (2022). Covid-19 vaccine effectiveness against the Omicron (B.1.1.529) variant. N. Engl. J. Med..

[3] Government of Ontario. All Ontarians 18+ eligible for COVID-19 booster appointments at three-month interval. https://news.ontario.ca/en/release/1001352/all-ontarians-18-eligible-for-covid-19-booster-appointments-at-three-month-interval (2021).

[4] Government of Ontario. Ontario expanding fourth-dose eligibility. https://news.ontario.ca/en/release/1001961/ontario-expanding-fourth-dose-eligibility (2022).

[5] Government of Ontario. Ontarians aged 18+ eligible for second booster shot. https://news.ontario.ca/en/release/1002191/ontarians-aged-18-eligible-for-second-booster-shot (2022).

[6] Grewal R (2022). Effectiveness of a fourth dose of covid-19 mRNA vaccine against the omicron variant among long term care residents in Ontario, Canada: test negative design study. BMJ.

[7] Government of Ontario. Ontarians aged 18+ eligible for bivalent COVID-19 booster dose. https://news.ontario.ca/en/release/1002277/ontarians-aged-18-eligible-for-bivalent-covid-19-booster-dose (2022).

[8] National Advisory Committee on Immunization. An Advisory Committee Statement (ACS) National Advisory Committee on Immunization (NACI): recommendations on the use of bivalent Omicron-containing mRNA COVID-19 vaccines. https://www.canada.ca/content/dam/phac-aspc/documents/services/immunization/national-advisory-committee-on-immunization-naci/recommendations-use-bivalent-Omicron-containing-mrna-covid-19-vaccines.pdf (2022).

[9] COVID-19 Immunity Task Force. Seroprevalence in Canada. https://www.covid19immunitytaskforce.ca/seroprevalence-in-canada/ (2022).

[10] Link-Gelles R (2022). Effectiveness of 2, 3, and 4 COVID-19 mRNA vaccine doses among immunocompetent adults during periods when SARS-CoV-2 Omicron BA.1 and BA.2/BA.2.12.1 sublineages predominated—VISION Network, 10 States, December 2021-June 2022. MMWR.

[11] Gazit S (2022). Short term, relative effectiveness of four doses versus three doses of BNT162b2 vaccine in people aged 60 years and older in Israel: retrospective, test negative, case-control study. BMJ.

[12] Magen O (2022). Fourth dose of BNT162b2 mRNA covid-19 vaccine in a nationwide setting. N. Engl. J. Med..

[13] Bar-On YM (2022). Protection by a fourth dose of BNT162b2 against Omicron in Israel. N. Engl. J. Med..

[14] UK Health and Security Agency. COVID-19 vaccine surveillance report: Week 40. https://assets.publishing.service.gov.uk/government/uploads/system/uploads/attachment_data/file/1109618/vaccine-surveillance-report-week-40.pdf (2022).

[15] Collie S (2022). Effectiveness and durability of the BNT162b2 vaccine against Omicron sublineages in South Africa. N. Engl. J. Med..

[16] Kislaya, I. et al. SARS-CoV-2 BA.5 vaccine breakthrough risk and severity compared with BA.2: a case-case and cohort study using Electronic Health Records in Portugal. Preprint at 10.1101/2022.07.25.22277996 (2022).

[17] Tseng, H. F. et al. Effectiveness of mRNA-1273 vaccination against SARS-CoV-2 omicron subvariants BA.1, BA.2, BA.2.12.1, BA.4, and BA.5. *Nat. Commun*. **14**, 189 (2023). 10.1038/s41467-023-35815-7 PMC9836332 36635284

[18] Surie D (2022). Effectiveness of monovalent mRNA vaccines against COVID-19-associated hospitalization among immunocompetent adults during BA.1/BA.2 and BA.4/BA.5 predominant periods of SARS-CoV-2 Omicron variant in the United States—IVY Network, 18 States, December 26, 2021-August 31, 2022. MMWR.

[19] Chalkias S (2022). A bivalent omicron-containing booster vaccine against Covid-19. N. Engl. J. Med..

[20] Collier, A. Y. et al. Immunogenicity of BA.5 Bivalent mRNA Vaccine Boosters. *N. Engl. J. Med.* **388**, 565–567 (2023). 10.1056/NEJMc2213948 PMC9847505 36630611

[21] Wang, Q. et al. Antibody Response to Omicron BA.4–BA.5 Bivalent Booster. *N. Engl. J. Med.* **388**, 567–569 (2023). 10.1056/NEJMc2213907 PMC9847504 36630643

[22] Government of Ontario. Updated eligibility for PCR testing and case and contact management guidance in Ontario. https://news.ontario.ca/en/backgrounder/1001387/updated-eligibility-for-pcr-testing-and-case-and-contact-management-guidance-in-ontario (2021).

[23] Chung H (2021). Effectiveness of BNT162b2 and mRNA-1273 covid-19 vaccines against symptomatic SARS-CoV-2 infection and severe covid-19 outcomes in Ontario, Canada: test negative design study. BMJ.

[24] Buchan SA (2022). Estimated effectiveness of COVID-19 vaccines against Omicron or Delta symptomatic infection and severe outcomes. JAMA Netw. Open.

[25] Ontario Agency for Health Protection and Promotion (Public Health Ontario). SARS-CoV-2 whole genome sequencing in Ontario, March 15, 2022. https://www.publichealthontario.ca/-/media/documents/ncov/epi/covid-19-sars-cov2-whole-genome-sequencing-epi-summary.pdf?sc_lang=en (2022).

[26] Ontario Agency for Health Protection and Promotion (Public Health Ontario). COVID-19 variant of concern omicron (B.1.1.529): risk assessment, January 12, 2022. https://www.publichealthontario.ca/-/media/documents/ncov/voc/2022/01/covid-19-omicron-b11529-risk-assessment-jan-12.pdf (2022).

[27] Ontario Agency for Health Protection and Promotion (Public Health Ontario). SARS-CoV-2 whole genome sequencing in Ontario, October 21, 2022. https://www.publichealthontario.ca/-/media/documents/ncov/epi/covid-19-sars-cov2-whole-genome-sequencing-epi-summary.pdf?sc_lang=en (2022).

[28] Ontario Agency for Health Protection and Promotion (Public Health Ontario). COVID-19 CCM case investigation data entry guide, version 2.0. (2022).

[29] Ontario Ministry of Health. COVID-19 vaccine booster recommendations. https://www.health.gov.on.ca/en/pro/programs/publichealth/coronavirus/docs/vaccine/COVID-19_vaccine_third_dose_recommendations.pdf (2022).

