# Author Correction: Effectiveness of mRNA COVID-19 vaccine booster doses against Omicron severe outcomes

**DOI:** 10.1038/s41467-023-37906-x

**Published:** 2023-04-11

**Authors:** Ramandip Grewal, Lena Nguyen, Sarah A. Buchan, Sarah E. Wilson, Sharifa Nasreen, Peter C. Austin, Kevin A. Brown, Deshayne B. Fell, Jonathan B. Gubbay, Kevin L. Schwartz, Mina Tadrous, Kumanan Wilson, Jeffrey C. Kwong

**Affiliations:** 1 Public Health Ontario, Toronto, ON Canada; 2 ICES, Toronto, ON Canada; 3 Dalla Lana School of Public Health, University of Toronto, Toronto, ON Canada; 4 Centre for Vaccine Preventable Diseases, University of Toronto, Toronto, ON Canada; 5 Institute of Health Policy, Management and Evaluation, University of Toronto, Toronto, ON Canada; 6 School of Epidemiology and Public Health, University of Ottawa, Ottawa, ON Canada; 7 Children’s Hospital of Eastern Ontario Research Institute, Ottawa, ON Canada; 8 Department of Laboratory Medicine and Pathobiology, University of Toronto, Toronto, ON Canada; 9 Women’s College Hospital, Toronto, ON Canada; 10 Leslie Dan Faculty of Pharmacy, University of Toronto, Toronto, ON Canada; 11 Department of Medicine, University of Ottawa, Ottawa, ON Canada; 12 Ottawa Hospital Research Institute, Ottawa, ON Canada; 13 Bruyere Research Institute, Ottawa, ON Canada; 14 Department of Family and Community Medicine, University of Toronto, Toronto, ON Canada; 15 University Health Network, Toronto, ON Canada

**Keywords:** Preventive medicine, Epidemiology, Vaccines, SARS-CoV-2

Correction to: *Nature Communications* 10.1038/s41467-023-36566-1, published online 07 March 2023

The original version of this Article contained errors in the column headings in the 2^nd^ row of Table 1, which stated ‘Omicron cases, *n* (%)’. The correct version of the 2^nd^ row of the 2^nd^, 5^th^, 8^th^ and 11^th^ columns now state ‘SARS-CoV-2 negative controls, *n* (%)^a^’ instead of the original ‘Omicron cases, *n* (%)’. The correct version of the 2^nd^ row of the 3^rd^, 6^th^, 9^th^, and 12^th^ columns now state ‘Omicron cases, *n* (%)’ instead of the original ‘SARS-CoV-2 negative controls, *n* (%)^a^’.

This has been corrected in both the PDF and HTML versions of the Article.

