# Supplementary material for: Effectiveness of mRNA COVID-19 vaccine booster doses against Omicron severe outcomes
Source: Nat Commun. 2023 Mar 7;14:1273. doi: 10.1038/s41467-023-36566-1 (PMC9990563; doi:10.1038/s41467-023-36566-1)
Supplement: Supplementary file 3 — Reporting Summary [file 41467_2023_36566_MOESM3_ESM.pdf]

nature portfolio

Corresponding author(s): Jeffrey C Kwong
Last updated by author(s): 2023/01/27

Reporting Summary

Nature Portfolio wishes to improve the reproducibility of the work that we publish. This form provides structure for consistency and transparency in reporting. For further information on Nature Portfolio policies, see our Editorial Policies and the Editorial Policy Checklist.

Statistics

For all statistical analyses, confirm that the following items are present in the figure legend, table legend, main text, or Methods section.

n/a	Confirmed	
☐	☒	The exact sample size (*n*) for each experimental group/condition, given as a discrete number and unit of measurement
☐	☒	A statement on whether measurements were taken from distinct samples or whether the same sample was measured repeatedly
☐	☒	The statistical test(s) used AND whether they are one- or two-sided<br>*Only common tests should be described solely by name; describe more complex techniques in the Methods section.*
☐	☒	A description of all covariates tested
☐	☒	A description of any assumptions or corrections, such as tests of normality and adjustment for multiple comparisons
☐	☒	A full description of the statistical parameters including central tendency (e.g. means) or other basic estimates (e.g. regression coefficient) AND variation (e.g. standard deviation) or associated estimates of uncertainty (e.g. confidence intervals)
☐	☒	For null hypothesis testing, the test statistic (e.g. *F*, *t*, *r*) with confidence intervals, effect sizes, degrees of freedom and *P* value noted<br>*Give P values as exact values whenever suitable.*
☒	☐	For Bayesian analysis, information on the choice of priors and Markov chain Monte Carlo settings
☒	☐	For hierarchical and complex designs, identification of the appropriate level for tests and full reporting of outcomes
☐	☒	Estimates of effect sizes (e.g. Cohen's *d*, Pearson's *r*), indicating how they were calculated

Our web collection on statistics for biologists contains articles on many of the points above.

Software and code

Policy information about availability of computer code

Data collection	*Provide a description of all commercial, open source and custom code used to collect the data in this study, specifying the version used OR state that no software was used.*
Data analysis	We used SAS version 9.1 (SAS Institute Inc., Cary, NC) for all analyses. The full dataset creation plan and underlying analytic code are available from the authors upon request, understanding that the computer programs may rely upon coding templates or macros that are unique to ICES and are therefore either inaccessible or may require modification.

For manuscripts utilizing custom algorithms or software that are central to the research but not yet described in published literature, software must be made available to editors and reviewers. We strongly encourage code deposition in a community repository (e.g. GitHub). See the Nature Portfolio guidelines for submitting code & software for further information.

Data

Policy information about availability of data

All manuscripts must include a data availability statement. This statement should provide the following information, where applicable:
- Accession codes, unique identifiers, or web links for publicly available datasets
- A description of any restrictions on data availability
- For clinical datasets or third party data, please ensure that the statement adheres to our policy

Pre-existing provincial SARS-CoV-2 laboratory testing, COVID-19 vaccination, and health administrative datasets were used for these analyses. These datasets were

linked using unique encoded identifiers and analyzed at ICES (formerly the Institute of Clinical Evaluative Sciences). The dataset from this study is held securely in coded form at ICES. While legal data sharing agreements between ICES and data providers (e.g., healthcare organizations and government) prohibit ICES from making the dataset publicly available, access may be granted to those who meet prespecified criteria for confidential access, available at www.ices.on.ca/DAS (email: das@ices.on.ca).

Human research participants

Policy information about studies involving human research participants and Sex and Gender in Research.

Reporting on sex and gender	We were limited to the variables available in the ICES administrative datasets. We report on sex (male and female) from the Registered Persons Database (RDP), but gender was not available. RDP contains information on persons registered under the Ontario Health Insurance Plan. Approximately 40% of the sample was male and 60% female.
Population characteristics	There were 11,160 cases of COVID-19 with Omicron-associated severe outcomes and 62,880 symptomatic test-negative controls (among 53,369 individuals) included in this analysis. Among adults aged 50-59 years, 60-69 years, 70-79 years, and 80+ years, respectively, 2%, 9%, 18%, and 20% received four doses of the COVID-19 vaccine. The mean age of cases was 76.7 years and for controls was 65.7 years. Among cases, 26.8% were from areas with the lowest household income quintile, 14% were from areas with the lowest essential workers quintile, and 25% lived in areas where 44-100% of individuals identified as a visible minority. Among controls, 20.9% were from areas with the lowest household income quintile, 15.4% were from areas with the lowest essential workers quintile, and 18.5% lived in areas where 44-100% of individuals identified as a visible minority.
Recruitment	Participants were not recruited for this study. This study was a secondary analysis using Ontario administrative data.
Ethics oversight	The use of the data in this study is authorized under section 45 of Ontario's Personal Health Information Protection Act, and does not require review by a research ethics board.

Note that full information on the approval of the study protocol must also be provided in the manuscript.

Field-specific reporting

Please select the one below that is the best fit for your research. If you are not sure, read the appropriate sections before making your selection.

☒ Life sciences ☐ Behavioural & social sciences ☐ Ecological, evolutionary & environmental sciences

For a reference copy of the document with all sections, see nature.com/documents/nr-reporting-summary-flat.pdf

Life sciences study design

All studies must disclose on these points even when the disclosure is negative.

Sample size	We included 11,160 Omicron-associated severe outcomes and 62,880 symptomatic test-negative controls. Sample size calculations were not conducted for this analysis. Sample sizes were determined based on the number of COVID-19 tested participants within our study period. Past studies we have conducted on COVID-19 vaccine effectiveness using administrative data in Ontario have supported the feasibility and sample sizes of subsequent vaccine effectiveness studies, such as this one.
Data exclusions	We included community-dwelling adults aged ≥50 years who had ≥1 reverse-transcription polymerase chain reaction (RT-PCR) test for SARS-CoV-2 between January 2, 2022 and October 1, 2022. We excluded immunocompromised individuals (n=11,514) and those who received a bivalent mRNA vaccine (n=2,433), Ad26.COV2 (n=83), or >1 dose of ChAdOx1-S (n=1,043) by the index date.
Replication	All code used to create the study cohort and analyze the data have been saved, and can be used to replicate this study.
Randomization	Since this was an observational study, randomization was not possible. We adjusted for confounding by using multivariable models and adjusting for sex, age (continuous), public health unit region, four area-level variables representing different socio-demographic characteristics (household income quintile, essential worker quintile, persons per dwelling quintile, self-identified visible minority quintile), influenza vaccination during 2019-2020 or 2020-2021 (proxy for health behaviours), SARS-CoV-2 infection >90 days prior, number of SARS-CoV-2 tests within 3 months prior to December 14, 2020 (proxy for healthcare workers), comorbidities, receipt of home care services, and week of test.
Blinding	This study is not a randomized trial. Additionally, we used existing data sources with no primary data collection. Therefore, blinding was not applicable. Nonetheless, the data used were at the individual level, exploration of covariates and analyses were done at the aggregate level and only the analyst had access to line-listed data. Therefore, it was not possible for investigators to know what group each individual was in.

Reporting for specific materials, systems and methods

We require information from authors about some types of materials, experimental systems and methods used in many studies. Here, indicate whether each material, system or method listed is relevant to your study. If you are not sure if a list item applies to your research, read the appropriate section before selecting a response.

Materials & experimental systems

n/a	Involved in the study
☒	☐ Antibodies
☒	☐ Eukaryotic cell lines
☒	☐ Palaeontology and archaeology
☒	☐ Animals and other organisms
☒	☐ Clinical data
☒	☐ Dual use research of concern

Methods

n/a	Involved in the study
☒	☐ ChIP-seq
☒	☐ Flow cytometry
☒	☐ MRI-based neuroimaging
